# Mechanically induced M2 macrophages are involved in bone remodeling of the midpalatal suture during palatal expansion

**DOI:** 10.1186/s40510-024-00529-z

**Published:** 2024-08-05

**Authors:** Lan Li, Mingrui Zhai, Chen Cheng, Shuyue Cui, Jixiao Wang, Zijie Zhang, Jiani Liu, Fulan Wei

**Affiliations:** Department of Orthodontics, School and Hospital of Stomatology, Cheeloo College of Medicine, Shandong Key Laboratory of Oral Tissue Regeneration & Shandong Engineering Laboratory for Dental Materials and Oral Tissue Regeneration & Shandong Provincial Clinical Research Center for Oral Diseases, Shandong University, No.44-1 Wenhua Road West, Shandong Jinan, 250012 China

**Keywords:** Palatal expansion, Macrophages, Bone remodeling, Immune

## Abstract

**Background:**

Palatal expansion is a common way of treating maxillary transverse deficiency. Under mechanical force, the midpalatal suture is expanded, causing local immune responses. This study aimed to determine whether macrophages participate in bone remodeling of the midpalatal suture during palatal expansion and the effects on bone remodeling.

**Methods:**

Palatal expansion model and macrophage depletion model were established. Micro-CT, histological staining, and immunohistochemical staining were used to investigate the changes in the number and phenotype of macrophages during palatal expansion as well as the effects on bone remodeling of the midpalatal suture. Additionally, the effect of mechanically induced M2 macrophages on palatal osteoblasts was also elucidated in vitro.

**Results:**

The number of macrophages increased significantly and polarized toward M2 phenotype with the increase of the expansion time, which was consistent with the trend of bone remodeling. After macrophage depletion, the function of osteoblasts and bone formation at the midpalatal suture were impaired during palatal expansion. In vitro, conditioned medium derived from M2 macrophages facilitated osteogenic differentiation of osteoblasts and decreased the RANKL/OPG ratio.

**Conclusions:**

Macrophages through polarizing toward M2 phenotype participated in midpalatal suture bone remodeling during palatal expansion, which may provide a new idea for promoting bone remodeling from the perspective of regulating macrophage polarization.

**Supplementary Information:**

The online version contains supplementary material available at 10.1186/s40510-024-00529-z.

## Background

Maxillary transverse deficiency is a common clinical malocclusion, and its main manifestations are crowded dentition, narrow dental arch, and unilateral or bilateral posterior crossbite [1, 2]. Currently, the most common treatment for maxillary transverse deficiency is palatal expansion, which applies a large orthopedic force in a short period to open the fibrous tissue connection at the midpalatal suture and eventually widen the maxillary dental arch [3]. Even after retention, relapse may occur despite the benefits of palatal expansion [4, 5], and insufficient bone formation is the main factor leading to relapse [6, 7]. Therefore, promoting bone remodeling during palatal expansion is critical to lower relapse rate, reduce retention time, and improve orthodontic treatment effects.

During palatal expansion, the midpalatal suture is stretched and enlarged by the expansion force, and cells proliferate and differentiate [8], causing local immune responses [9, 10]. Macrophages, a crucial part of the innate immune system [11], are essential for maintaining osteoblast function and wound healing responses [12, 13]. Macrophages have considerable diversity and plasticity, and they can polarize toward M1 or M2 phenotypes. Tumor necrosis factor-α (TNF-α), inducible nitric oxide synthase (INOS), and other inflammatory mediators are produced by M1 macrophages, which mediate inflammation responses; however, M2 macrophages secrete anti-inflammatory cytokines, including interleukin-10 (IL-10) and transforming growth factor-β (TGF-β), etc [14]. Recently, it has been discovered that macrophages were responsive to mechanical stimuli, which can alter their phenotypes and then trigger them to secrete various cytokines to regulate the local microenvironment [15]. He et al. [16, 17] found that macrophages polarized toward the M1 phenotype to promote orthodontic tooth movement. Liang et al. [18] reported that macrophages responded to mechanical stretch and polarized toward M2 phenotype to achieve bone regeneration, suggesting that modulation of the macrophage phenotype may promote the bone remodeling process.

Thus, we hypothesized that macrophages may have a vital role in bone remodeling during palatal expansion. We established palatal expansion model and macrophage depletion model in rats in this study to determine whether macrophages participate in mechanical force-induced bone remodeling of the midpalatal suture, and further explore the possible mechanism, which may provide ideas for orthodontists to search for new targets in order to improve the efficiency of palatal expansion and lower the relapse rate.

## Materials and methods

### Animals

Six-week-old male Wistar rats (Pengyue, China) were used in our study. Animal experiments were approved by the Ethics Committee of the School of Stomatology, Shandong University (No. 20201102). The rats were kept in plastic cages with the temperature of 25 °C. During the experiment, the rats were fed rodent food and weighed every other day.

### Palatal expansion procedure

**Fig. 1:**
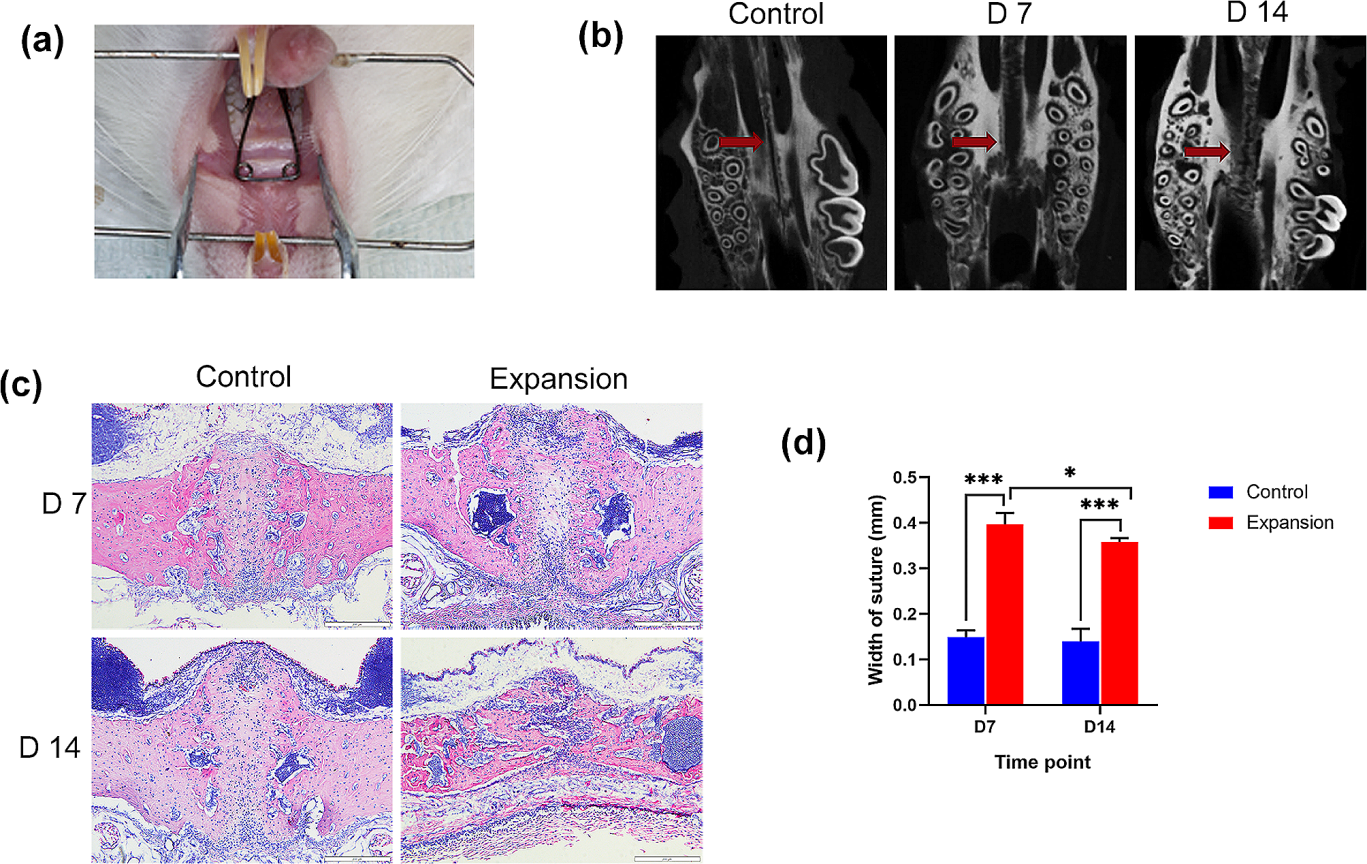
Changes of midpalatal suture during palatal expansion in rats. (**a**) Palatal expansion models built in Wistar rats. (**b**) Micro-CT reconstruction images of midpalatal suture for the control group and expansion group on days 7 and 14. (**c**) H&E staining of the midpalatal suture of the control group and expansion group on days 7 and 14, scale bars: 200 μm. (**d**) The width of the midpalatal suture of the expansion and control groups on days 7 and 14. The red arrow indicated the midpalatal suture. Data indicate the mean ± S.D, *P < 0.05, ***P < 0.001

The expanders were made using 0.014-inch Australian wire (AJ Wilcock Pty Ltd, Australia), with the expansion force of approximately 100 ± 10 g. The expander was placed between bilateral maxillary molars (Fig. 1a), and the retention was strengthened with flowable resin (3M ESPE, USA). During the operation, the rats’ body temperature was maintained and their vital signs were carefully monitored until they completely awoke. The control group was without palatal expansion. On days 7 and 14, all rats were executed and their maxillae were collected.

### In vivo depletion of macrophages

The experimental group received intravenous administration of clodronate liposomes (CLOD; 15 mg/kg, Clodronate Liposomes, Netherlands) to deplete macrophages [19], while phosphate buffered saline (PBS; Basal Media, China) was given to the control group [20, 21]. All rats received injections on the day that the expanders were installed and then every 4 days until they were sacrificed on days 7 and 14. The efficiency of macrophage depletion was verified by flow cytometry and immunohistochemical staining [22, 23].

### Flow cytometry

Bone marrow cells from the tibia were treated with anti-F4/80-fluorescein isothiocyanate antibody (Bioss, China) for 40 min in ice. PBS was utilized as the control. The BD Accuri C6 Plus Software was used to assess the percentages of positive cells (version 1.0.23.1, BD Biosciences, USA).

### Micro-computed tomography (Micro-CT) analysis

The samples were scanned using the micro-CT system (Quantum GX2, PerkinElmer, Japan) with a standard acquisition protocol (90 kV, 88 µA, 72 μm voxel size). CTAn software (version 1.17.7.2, Skyscan, Bruker microCT, Belgium) was used to reconstruct images and data measurements. The midpalatal suture width between bilateral maxillary first molars, bone mineral density (BMD), and bone volume fraction (BV/TV) of new bone were evaluated by a single examiner.

### Histological and tartrate-resistant acid phosphatase (TRAP) staining

After being demineralized with 10% ethylenediaminetetraacetic acid, the samples were dehydrated and embedded in paraffin and then cut into 5 μm sections. The midpalatal suture morphology was assessed by hematoxylin and eosin (H&E) staining (Solarbio, China). In order to quantify osteoclasts, TRAP staining was performed according to the kit’s instructions (Solarbio, China).

### Immunohistochemistry

Immunohistochemistry was performed on the primary antibodies against F4/80 (Bioss, China), OCN (Bioss, China), ALP (HuaBio, China), CD206 (Proteintech, USA), INOS (Proteintech, USA), OPG (Bioss, China), and RANKL (Proteintech, USA). Briefly, sections were processed with 0.1% trypsin for 30 min at 37°C (Solarbio, China), and then with 3% hydrogen peroxide for 20 min. Following that, 5% goat serum was added and incubated for one hour. Sections were treated with the secondary antibodies after the primary antibodies were added overnight at 4°C, followed by 3,3’-diaminobenzidine to detect immunoactivity (Zhongshan Golden Bridge Biotechnology, China). Ultimately, hematoxylin was used to counterstain the sections. The outcomes were assessed utilizing the ImageJ software (version 1.51k, NIH, USA). For each slide, at least three fields of view were randomly assessed by a single examiner, and the outcomes then were averaged across all three fields of view.

### Cell isolation and culture

The palatal bones of 3 to 5-day-old Wistar rats were aseptically removed and cut into 1 mm bone pieces before being processed with trypsin for 20 min at 37 °C. Then, the initial digest was discarded and bone pieces were digested again with 0.1% collagenase II (Roche, Japan) for 60 min. The secondary digested cells and bone fragments were collected and seeded in culture flasks containing 20% fetal bovine serum (FBS; Lonsa Science SRL, Uruguay) and 2% penicillin-streptomycin (Biosharp, China) in the alpha-modification of eagle’s medium (α-MEM, Basal Media, China) at 37 ℃ in 5% CO2. One week later, the primary palatal osteoblasts were passaged.

The RAW264.7 cells were cultured in α-MEM containing 10% FBS, which was maintained at 37 °C in 5% CO2.

### Application of mechanical force in vitro

RAW264.7 cells were cultured at a density of 1.5 × 10^5^ per well on Flexcell amino-silicon bottom six-well culture plates that were coated with collagen I solution (Corning, USA). The Flexcell-FX-5000-Tension System was utilized to exert mechanical force (Flexcell International Corporation, USA). Cells were stimulated with 10% stretch for 48 h at 0.5 Hz, and the supernatant was collected to assess IL-10 and TNF-α levels using enzyme-linked immunosorbent assay (ELISA; Elabscience, China). The control group was without applying mechanical stretch.

### Immunofluorescence staining

Cells were fixed with 4% paraformaldehyde (Solarbio, China) for 30 min, then permeabilized by adding 0.5% Triton X-100 (Solarbio, China), followed by blocking with 5% goat serum. The primary antibodies, including anti-CD206 (Proteintech, USA) and anti-INOS (Proteintech, USA), were then incubated overnight at 4 °C, subsequently undergoing one hour incubation with the secondary antibodies (Proteintech, USA). Finally, cell nuclei were stained using 4’,6-diamidino-2-phenylindole (DAPI; Solarbio, China) for 10 min. An inverted confocal microscope was used to take the images (Leica, Germany), and the outcomes were analyzed by a single examiner.

### Osteogenic induction, ALP, and alizarin red staining

Palatal osteoblasts were cultured in 12-well plates. Following a 24-hour period of adhesion, the medium was subsequently substituted for the osteogenic-inducing medium that consisted of α-MEM and the collected macrophage supernatant in a 1:1 ratio, along with 50 µg/mL ascorbic acid and 10 mM β-glycerophosphate (Sigma, USA). The osteogenic-inducing medium was changed at intervals of 3 to 4 days. The ALP staining kit was utilized to carry out ALP staining following a 7-day culture (Biotime, China). Additionally, ALP activity kit was employed to measure its activity (Nanjing Jiancheng Bioengineering Institute, China). Staining with 2% alizarin red was conducted on palatal osteoblasts after 28 days (pH = 4.2) (Solarbio, China). The mineralized matrix was dissolved by adding 10% cetylpyridinium chloride (Solarbio, China), and then by using spectrophotometry at 562 nm, the mineralized matrix’s quantity was measured.

### Quantitative real‑time PCR (qRT-PCR)

Total RNA was extracted by AG RNAex Pro Reagent (AG, China) and then reverse transcribed into cDNA using the Evo M-MLV RT Mix Kit (AG, China). SYBR Green Premix Pro Taq HS qPCR Kit II (AG, China) was used to conduct qRT-PCR for the following genes: INOS, TNF-α, IL-10, TGF-β, ALP, COL1, RANKL, and OPG. The comparative 2^−ΔΔCt^ approach was utilized to establish relative quantification. Table 1 lists the primers that were employed in this study.

**Table 1:** qRT-PCR primer sequence

Target gene	Forward primer	Reverse primer
TGF-β	AGCTGCGCTTGCAGAGATTA	AGCCCTGTATTCCGTCTCCT
IL-10	GCTCTTGCACTACCAAAGCC	CTGCTGATCCTCATGCCAGT
TNF-α	AGCCGATGGGTTGTACCTTG	ATAGCAAATCGGCTGACGGT
INOS	CCTGCTTTGTGCGAAGTGTC	CCCAAACACCAAGCTCATGC
ALP	ACAACACCAACGCTCAGGTC	GTGACCTCGTTCCCCTGAGT
COL1	CACTGCAAGAACAGCGTAGC	AAGTTCCGGTGTGACTCGTG
RANKL	TACTTTCGAGCGCAGATGGAT	AGTGCTTCTGTGTCTTCGCT
OPG	CGGATGGGTTCTTCTCAGGT	TCGCACAGGGTGACATCTATTC
GAPDH (rat)	TCTCTGCTCCTCCCTGTTCT	ATCCGTTCACACCGACCTTC
GAPDH (mouse)	TGTCTCCTGCGACTTCAACA	GGTGGTCCAGGGTTTCTTACT

### Western blotting

The western blot procedure was carried out as described in our previous studies [24]. Cells were lysed using the RIPA reagent (Solarbio, China), which contains 1% PMSF (Solarbio, China). The lysed samples were transferred using polyvinylidene fluoride membranes (PVDF; Millipore, USA) after being loaded onto 10% sodium dodecyl sulfate-polyacrylamide electrophoresis gels to separate them. Prior to incubating the membranes with primary antibodies overnight at 4 °C, they were blocked for one hour in 5% skimmed milk. The primary antibodies were anti-GAPDH (Proteintech, USA), anti-ALP (Proteintech, USA), and anti-COL1 (Proteintech, USA). The immunoreactive bands were detected with an ECL chromogenic substrate (Millipore, USA) after the second antibodies were incubated for one hour (Proteintech, USA). A single examiner quantified the densitometric data using ImageJ software (version 1.51k, NIH, USA). GAPDH was employed as the control in the immunoreactive bands analysis.

### Statistical analysis

GraphPad Prism (version 8.0.2, GraphPad program Inc., USA) was employed to conduct all analyses. All normally distributed data were presented as the means ± standard deviation of at least three independent samples. The differences between the two groups were analyzed using Student’s t-tests. Three or more groups were analyzed using a one-way analysis of variance (ANOVA) with Bonferroni’s post hoc test. Statistical significance was defined as *p* < 0.05. All micro-CT and histological measurements were reassessed after one month by the same examiner in order to analyze the reliability of the measurements. The intra-class correlation coefficient was performed to test the intra-examiner reliability.

## Results

### Macrophages participated in bone remodeling of the midpalatal suture during palatal expansion

**Fig. 2:**
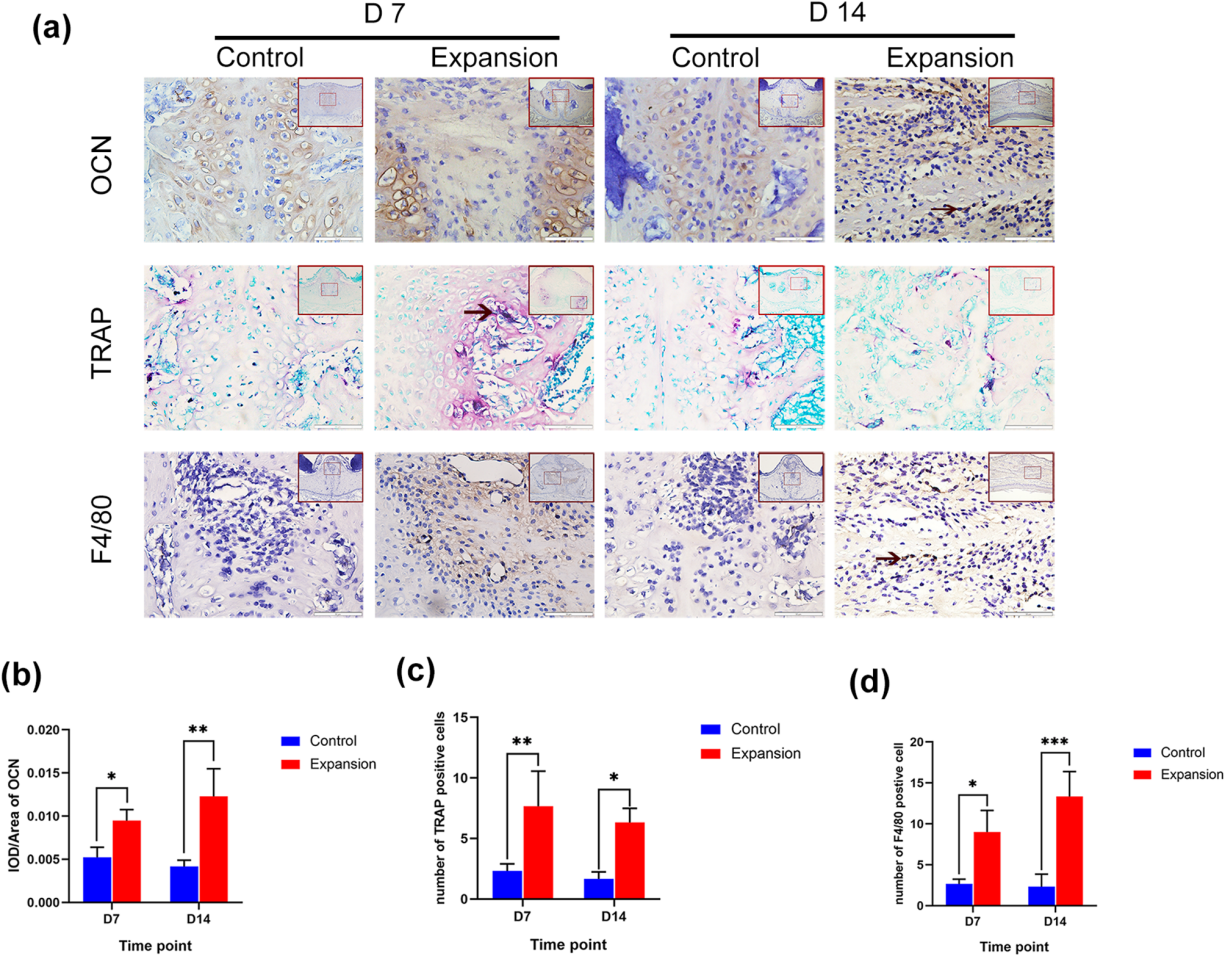
Changes of macrophages during the bone remodeling of midpalatal suture after force application. (**a**) TRAP staining, OCN, and F4/80 immunohistochemical staining of midpalatal suture of the control group and expansion group on days 7 and 14. (**b**-**d**) OCN, TRAP, and F4/80 quantification analysis of midpalatal suture of the control group and expansion group on days 7 and 14. Large red boxed areas show 100 × magnification views. Large pictures showed 400 × magnification views of the small red boxes. Scale bars: 200 μm (100×); 50 μm (400×). Data indicate the mean ± S.D, *P < 0.05, **P < 0.01, ***P < 0.001. IOD, integrated optical density. The arrow indicated the positive cells

The rats’ body weight in the control group exhibited a constant increase over the course of the experiment. However, because the expanders were installed, there was a considerable drop in the rats’ body weight in the expansion group for the first two days, but a steady increase starting on the third day (Fig. S1a), which was in line with previous studies [1, 25]. Compared with the 7-day expansion group, new bone formation was obviously observed in the 14-day expansion group (Fig. 1b). The same phenomenon was also observed by H&E staining (Fig. 1c). The midpalatal suture was successfully enlarged, as shown by the fact that on days 7 and 14, the width was noticeably greater in the expansion group (Fig. 1d). In addition, it was found that periosteal cells migrated into the midpalatal suture, with their number gradually increasing during palatal expansion (Fig. 1c). The activity of osteoblasts was also observed since palatal expansion is a bone remodeling process involving the balance between osteoblasts and osteoclasts. It was noticeable on days 7 and 14 that the expansion group had higher OCN expression and more TRAP-positive cells than the control group (Fig. 2a-c).

To evaluate the dynamic changes of macrophages during palatal expansion, F4/80 immunohistochemical staining was performed. We found that F4/80-positive macrophages were distributed around the blood vessels at the midpalatal suture and the palatal bone marrow cavity. On days 7 and 14, the expansion group had considerably more macrophages than the control group. Additionally, we discovered that macrophages increased significantly on day 14 of palatal expansion when bone formation was evident at the midpalatal suture. Apart from the above distribution locations, macrophages were also distributed on the new bone surface, adjacent to osteoblasts (Fig. 2a, d), which further suggested that macrophages may be closely related to osteoblasts.

### Macrophage depletion by injection of clodronate liposomes inhibited bone remodeling during palatal expansion

**Fig. 3:**
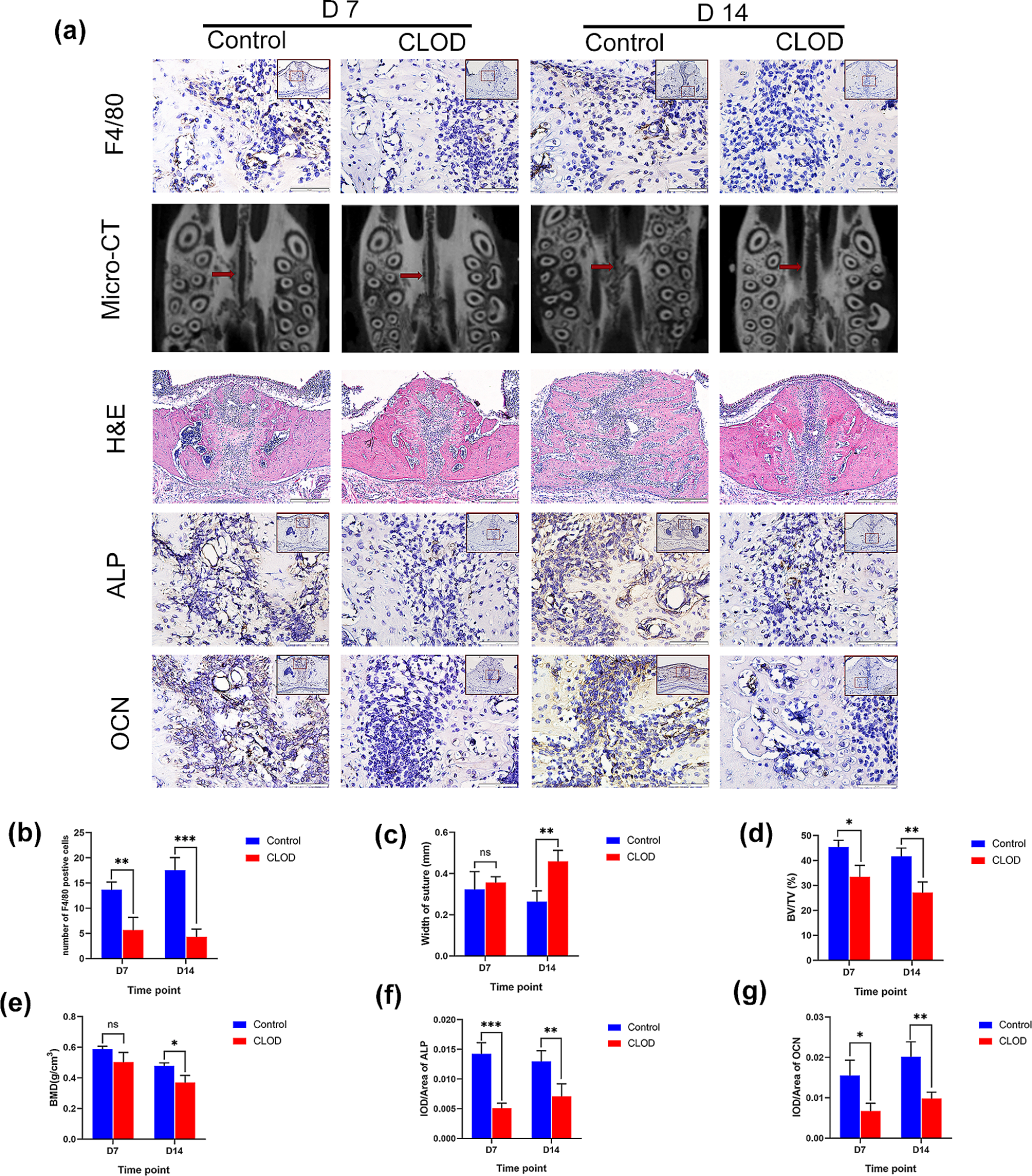
Effect of macrophage depletion by injection of clodronate liposomes on bone formation during palatal expansion. (**a**) Micro-CT reconstruction images, H&E staining, F4/80, ALP, and OCN immunohistochemical staining on days 7 and 14. (**b**) F4/80 quantification analysis of midpalatal suture of the control group and CLOD group on days 7 and 14. (**c**) The width of the midpalatal suture measured by Micro-CT. (**d**-**e**) Bone volume fraction (BV/TV) and bone mineral density (BMD) of midpalatal suture of the control group and the CLOD group on days 7 and 14. (**f**, **g**) ALP and OCN quantification analysis of midpalatal suture of the control group and CLOD group on days 7 and 14. Large red boxed areas show 100 × magnification views, Large pictures showed 400 × magnification views of the small red boxes. Scale bars: 200 μm (100×); 50 μm (400×). Data indicate the mean ± S.D, *P < 0.05, **P < 0.01, ***P < 0.001, ns, not significant (*P* > 0.05). IOD, integrated optical density; CLOD, clodronate liposomes

To further investigate the effect of macrophages on bone remodeling of the midpalatal suture, we constructed macrophage depletion model using clodronate liposomes. Rats in the CLOD group consistently had lower body weights than those in the control group (Fig. S1b). After injection of clodronate liposomes to deplete macrophages, immunohistochemical staining and flow cytometry results showed the number of F4/80-positive macrophages decreased significantly on days 7 and 14 (Fig. 3a, b and Fig. S2).

H&E staining and micro-CT images revealed that bone formation considerably decreased following macrophage depletion (Fig. 3a). Furthermore, when compared to the control group, the midpalatal suture width was also noticeably wider in the CLOD group on 14 days (Fig. 3c). The BMD and BV/TV correspondingly decreased on days 7 and 14 in the CLOD group (Fig. 3d, e). ALP and OCN were also substantially elevated in the control group (Fig. 3a, f, g), whereas they were only weakly expressed in the CLOD group. The above findings suggested that inhibiting the function of macrophages might hinder bone formation during palatal expansion.

**Fig. 4:**
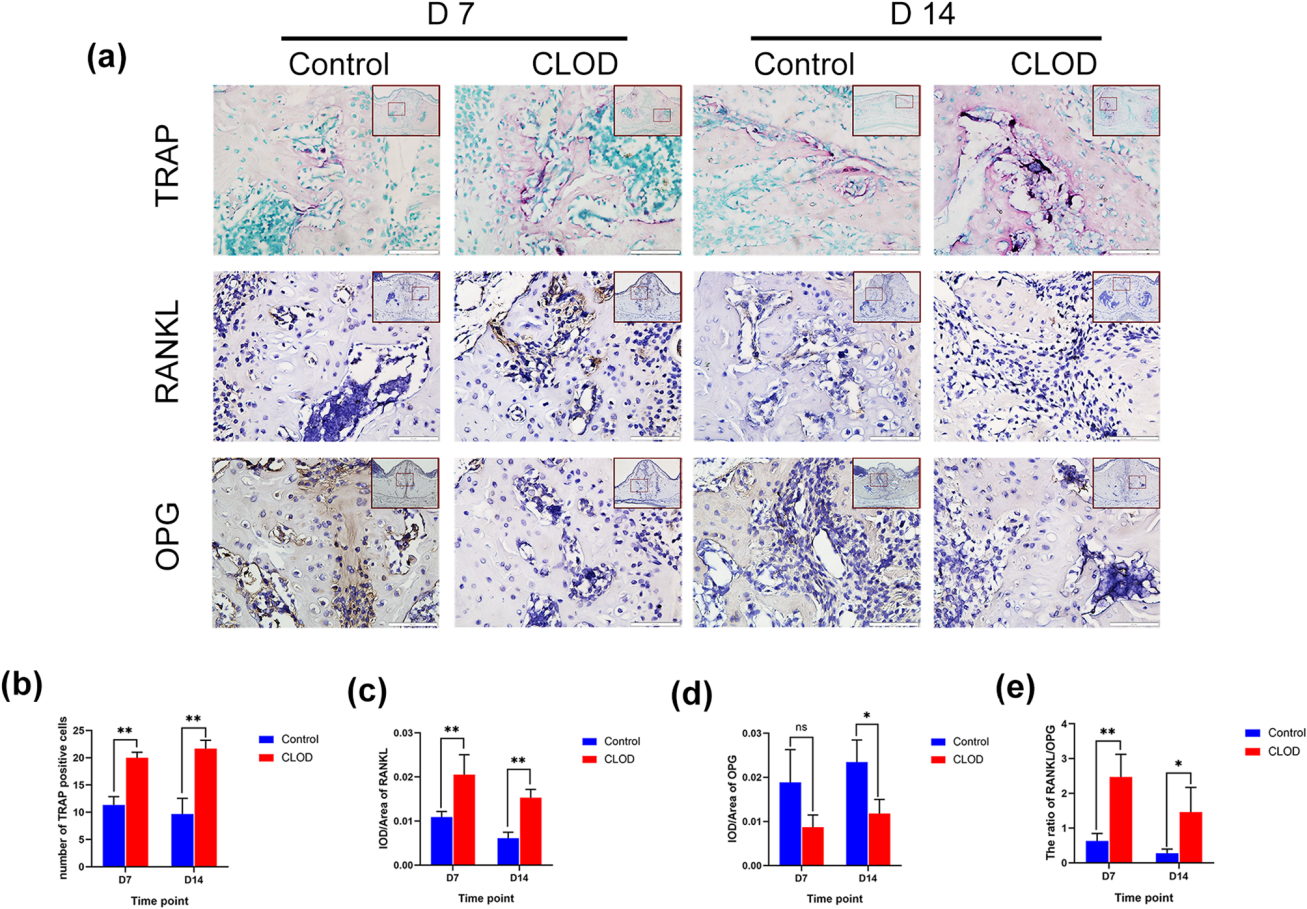
Effect of macrophage depletion by injection of clodronate liposomes on bone resorption during palatal expansion. (**a**) RANKL and OPG immunohistochemical staining, TRAP staining of midpalatal suture of the control group and the CLOD group on days 7 and 14. (**b**-**d**) TRAP, RANKL, and OPG quantification analysis of midpalatal suture of the control group and the CLOD group on days 7 and 14. (**e**) The ratio of RANKL/OPG. Large red boxed areas show 100 × magnification views. Large pictures showed 400 × magnification views of the small red boxes. Scale bars: 200 μm (100×); 50 μm (400×). Data indicate the mean ± S.D, *P < 0.05, **P < 0.01, ns, not significant (*P* > 0.05). IOD, integrated optical density; CLOD, clodronate liposomes

Osteoclasts by TRAP staining were also observed to evaluate the bone remodeling status of both groups. The results showed that on days 7 and 14, there were significantly more osteoclasts in the CLOD group (Fig. 4a, b). Additionally, the RANKL/OPG ratio exhibits an equilibrium between bone formation and resorption. A higher RANKL/OPG ratio indicates that bone resorption is dominant, while a lower ratio indicates bone formation. The CLOD group exhibited a markedly higher RANKL/OPG ratio than the control group (Fig. 4a, c, d, e), revealing that macrophage depletion could inhibit bone remodeling. All the findings confirmed that macrophages were important in the midpalatal suture bone remodeling triggered by mechanical force.

### M2 macrophages responded to mechanical force in vivo and in vitro

**Fig. 5:**
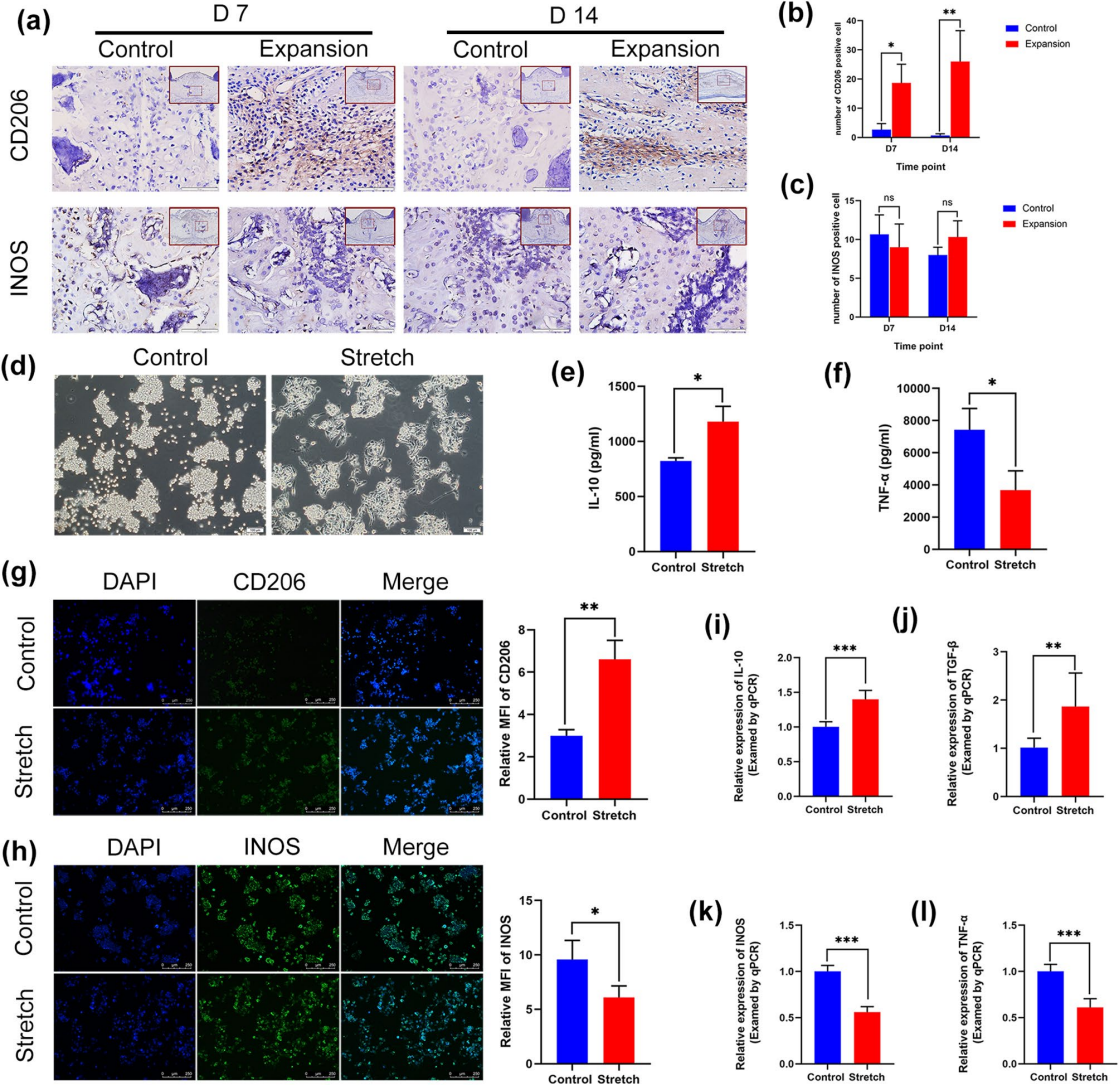
M2 macrophages responded to mechanical force in vivo and in vitro. (**a**-**c**) CD206 and INOS immunohistochemical staining and quantification analysis of midpalatal suture in the control group and the expansion group on days 7 and 14. (**d**) Representative images of control and stretched macrophages. (**e**-**f**) IL-10 and TNF-α concentrations in the supernatant of the control group and the stretch group. (**g**-**h**) Immunofluorescence staining and quantification analysis for CD206 and INOS in the control group and the stretch group. (**i**-**l**) The expression level of IL-10, TGF-β, INOS, and TNF-α was evaluated by qRT-PCR in the control group and the stretch group. Large red boxed areas show 100 × magnification views. Large pictures showed 400 × magnification views of the small red boxes. Scale bars: 200 μm (100×); 50 μm (400×). Data indicate the mean ± S.D, *P < 0.05; **P < 0.01, ***P < 0.001, ns, not significant (*P* > 0.05). MFI, mean fluorescence intensity

To understand how macrophages participate in bone remodeling of the midpalatal suture, we further determined the phenotypic changes of macrophages during palatal expansion. Immunohistochemical staining for CD206 and INOS was performed. On days 7 and 14, there was a considerable rise in CD206-positive macrophages in the expansion group (Fig. 5a, b). However, no discernible difference was found in INOS-positive macrophages between the two groups (Fig. 5a, c). These findings showed that macrophages might participate in bone remodeling of the midpalatal suture through polarization toward M2 phenotype in vivo.

Then, to explore the response of macrophages to mechanical forces in vitro, we applied mechanical stretch to RAW264.7 cells. The results revealed that the macrophages in the stretch group displayed irregular extended morphology (Fig. 5d). The ELISA kit was employed to identify the content of M1/M2-related cytokines in the supernatant. IL-10 rose notably in the stretch group (Fig. 5e), but TNF- α was reduced (Fig. 5f). According to the immunofluorescence results, the stretch group’s expression of CD206 was higher (Fig. 5g) and that of INOS was lower in comparison to the control group (Fig. 5h). Additionally, M1/M2-related genes’ expression was determined using qRT-PCR. It was discovered that IL-10 and TGF-β (M2-related genes) were enhanced in the stretch group (Fig. 5i, j), while INOS and TNF-α (M1-related genes) were decreased (Fig. 5k, l). Collectively, these findings suggested that appropriate mechanical force could induce macrophage polarization toward M2 phenotype rather than M1 phenotype in vitro.

**Conditioned medium from force-induced M2 macrophages can promote palatal osteoblasts osteogenic differentiation and lower RANKL/OPG ratio**.

**Fig. 6:**
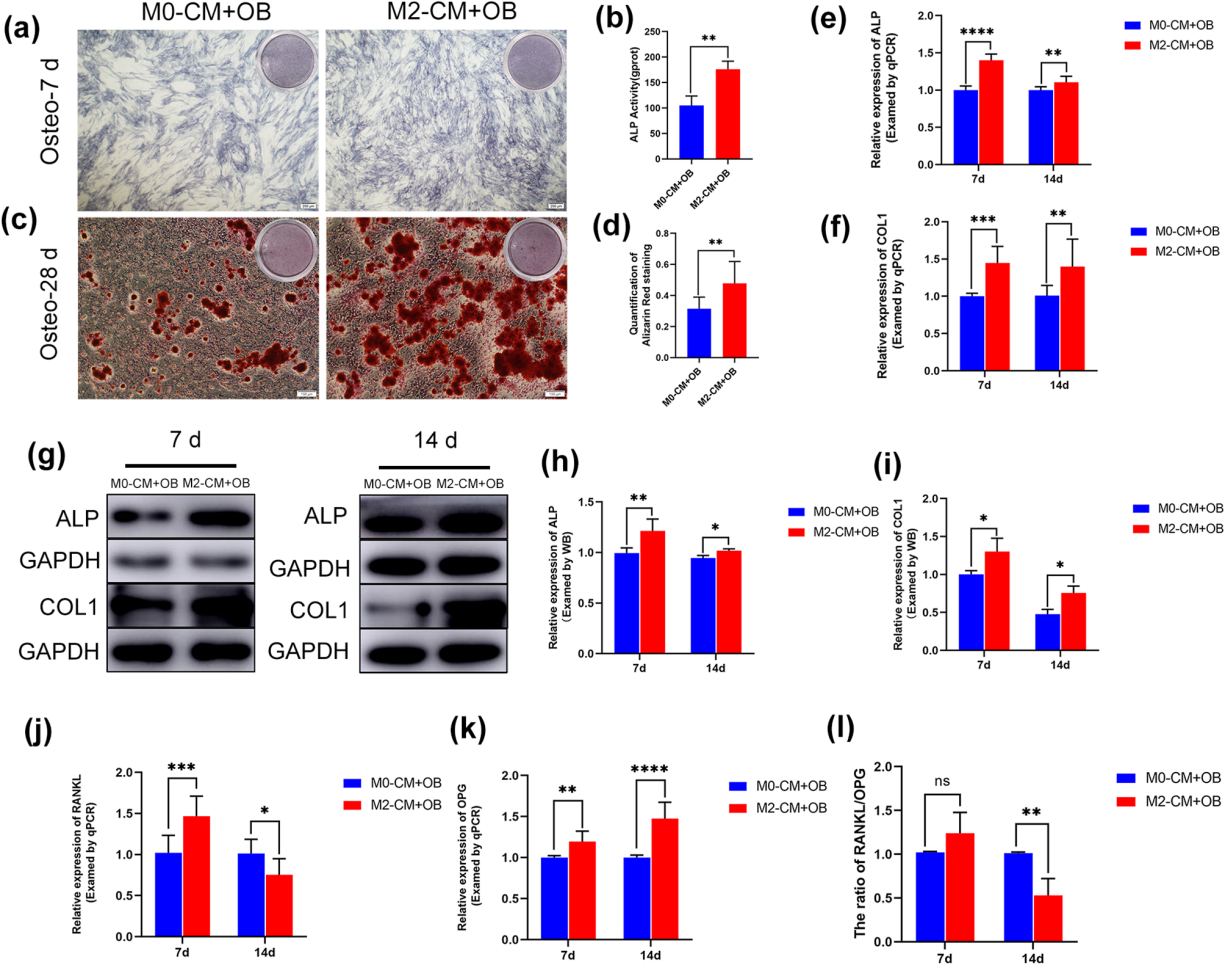
Conditioned medium from force-induced M2 macrophages can promote palatal osteoblasts osteogenic differentiation and lower RANKL/OPG ratio. (**a**-**b**) ALP staining and quantitative analysis at 7 days in both groups. (**c**-**d**) Alizarin red staining and quantitative analysis at 28 days in both groups. (**e**-**f**) The relative gene expression of ALP and COL1 after osteogenic induction for 7 and 14 days in both groups. (**g**-**i**) The protein level of ALP and COL1 in both groups after osteogenic induction for 7 and 14 days. (**j**-**k**) The relative gene expression of RANKL and OPG after osteogenic induction for 7 and 14 days. (**l**) The ratio of RANKL/OPG after osteogenic induction for 7 and 14 days. Scale bar: 100/200µm. Data indicate the mean ± S.D, *P < 0.05, **P < 0.01, ***P < 0.001, ****P < 0.0001, ns, not significant (*P* > 0.05). M0-CM, M0 macrophage-derived conditioned medium; M2-CM, M2 macrophage-derived conditioned medium; OB, osteoblast

To determine the effect of force-induced M2 macrophages on palatal osteoblasts, palatal osteoblasts were induced in osteogenic induction medium containing conditioned medium from force-induced M2 macrophages supernatant (M2-CM) and non-polarized M0 macrophages supernatant (M0-CM). ALP activity and mineralized nodule formation capacity increased in osteoblasts induced by M2-CM compared to M0-CM (Fig. 6a-d). qRT-PCR revealed that ALP and COL1 expressions considerably increased in palatal osteoblasts cultured using M2-CM (Fig. 6e, f), which coincided with the results of the western blot (Fig. 6g-i). These findings indicated that M2 macrophage-derived conditioned medium can promote osteogenic differentiation of osteoblasts.

RANKL and OPG, which are produced by osteoblasts, are essential molecules that regulate bone remodeling [26], so we further investigated their expression in osteoblasts induced by macrophage-derived conditioned medium on 7 and 14 days in both groups. qRT-PCR showed that RANKL expression in osteoblasts induced by M2-CM increased on day 7 and reduced on day 14, while OPG expression increased on days 7 and 14. Moreover, the RANKL/OPG ratio did not change significantly on day 7, but decreased on day 14 of osteogenic induction (Fig. 6j-l), suggesting that M2 macrophages may indirectly affect osteoclasts through the RANKL/OPG pathway.

## Discussion

The maxillary dental arch may narrow again as a result of inadequate bone formation during palatal expansion, which could cause the deformity to recur. Orthodontic clinical practice typically extends the course of treatment to achieve stability of the palatal expansion in order to reduce the likelihood of relapse, but this may inconvenience patients by making it harder for them to maintain good oral hygiene. Therefore, it is crucial for orthodontic clinical practice to identify effective means of promoting bone remodeling in the midpalatal suture. Although current research on promoting bone remodeling focuses on various aspects, such as drugs [27], hormones [28], and physical stimulation [29], few studies have explored ways to modulate bone remodeling from an immunological perspective. Our study provided evidence that M2 macrophages participated in bone remodeling during palatal expansion and that bone formation at the midpalatal suture is reduced after macrophage depletion.

The midpalatal suture width significantly widened on 7 days in the expansion group but decreased on 14 days, possibly due to new bone formation at the late stage, which was consistent with the previous studies [8, 30]. The OCN immunohistochemical results further confirmed the enhancement of osteogenesis at the midpalatal suture with increasing time of palatal expansion. However, TRAP-positive cells decreased on 14 days. This may be a result of a disruption of the dynamic balance between osteogenesis and osteoclastogenesis, with enhanced osteogenesis and a corresponding weakening of osteoclastogenesis, which is consistent with previous studies [9, 31].

Macrophages can polarize toward specific phenotypes to promote tissue regeneration when stimulated by mechanical forces [15]. Ding et al. [32] found that macrophages were consistently maintained at a high-level during skin expansion, with M1 macrophages predominating early and M2 macrophages prevailing later. Chu et al. [33] and Liang et al. [18] discovered that during hair and zygomatic bone expansion, macrophages polarized toward the M2 phenotype and promoted hair and bone regeneration, which is consistent with our findings that M2 macrophages increased during palatal expansion corresponding to the trend of bone remodeling at the midpalatal suture. The difference between our results and those of Ding et al. [32] may be due to the installation of the expansion device after skin incision in their experiments, which may have led to an increase in M1 macrophages early during skin expansion.

Macrophage depletion has been found to inhibit regeneration in several organs and tissues [34–36], impaired fracture healing, and reduced localized bone formation [37]. In our study, we discovered that bone formation at the midpalatal suture was severely diminished at 14 days after macrophage depletion, further confirming the importance of macrophages in bone regeneration. Mineralization capacity and the expression of genes associated with osteogenesis of osteoblasts were found to be significantly reduced after the removal of macrophages from calvarial preparations [13], and macrophage depletion in vivo was accompanied by a decrease in osteoblasts and bone formation [38, 39]. In our study, ALP and OCN expression at the midpalatal suture were markedly decreased after macrophage depletion, suggesting that macrophage depletion may affect the osteogenic capacity of osteoblasts.

Mechanical force-induced macrophage polarization toward specific phenotypes is highly dependent on the duration of applied force, magnitude, and frequency, etc. Dong et al. [40] discovered that 5% stretch triggered polarization of macrophages toward the M2 phenotype, while 15% stretch induced polarization of the M1 phenotype. Liang et al. [18] found that M1 and M2 macrophages induced by 5% stretch had no appreciable variations compared with the control group. However, a 10% stretch can promote polarization of M2 macrophages in vitro, which was in agreement with our results. Previous studies found that M2 macrophages could facilitate osteogenic differentiation of mesenchymal stem cells [41, 42]. Similarly, our study discovered that stretch-induced M2 macrophages-derived conditioned medium could enhance osteogenic differentiation in palatal osteoblasts, and significantly decrease the ratio of RANKL/OPG, consistent with the previous findings [43].

However, our study has some limitations. We did not explore the changes in macrophages during the retention period after palatal expansion, which might require to be confirmed by further research. In addition, there is a need for research to determine the underlying mechanisms that how polarized macrophages regulate palatal osteoblasts at the midpalatal suture, as well as the mechanism of M2 macrophage activation in response to mechanical forces during palatal expansion.

## Conclusion

In summary, this study revealed that macrophages through polarizing toward M2 phenotype participated in mechanical force-induced midpalatal suture bone remodeling, which may provide a new idea for facilitating bone remodeling during palatal expansion from the perspective of regulating macrophage polarization.

### Electronic supplementary material

Below is the link to the electronic supplementary material.

Supplementary Material 1

## Data Availability

Data sharing is not applicable to this article as no datasets were generated or analysed during the current study.

## Abbreviations

Micro-CT: Micro-computed tomography
CLOD: Clodronate liposomes
BV/TV: Bone volume fraction
GAPDH: Glyceraldehyde-3-phosphate dehydrogenase
BMD: Bone mineral density
MFI: Mean fluorescence intensity
M0-CM: M0 macrophage-derived conditioned medium
M2-CM: M2 macrophage-derived conditioned medium
IOD: Integrated optical density
OB: Osteoblast
