# Supplementary material for: Mechanically induced M2 macrophages are involved in bone remodeling of the midpalatal suture during palatal expansion
Source: Prog Orthod. 2024 Aug 5;25:30. doi: 10.1186/s40510-024-00529-z (PMC11298508; doi:10.1186/s40510-024-00529-z)
Supplement: Supplementary file 1 — Supplementary Material 1 [file 40510_2024_529_MOESM1_ESM.docx]

**Supplementary Materials**

**
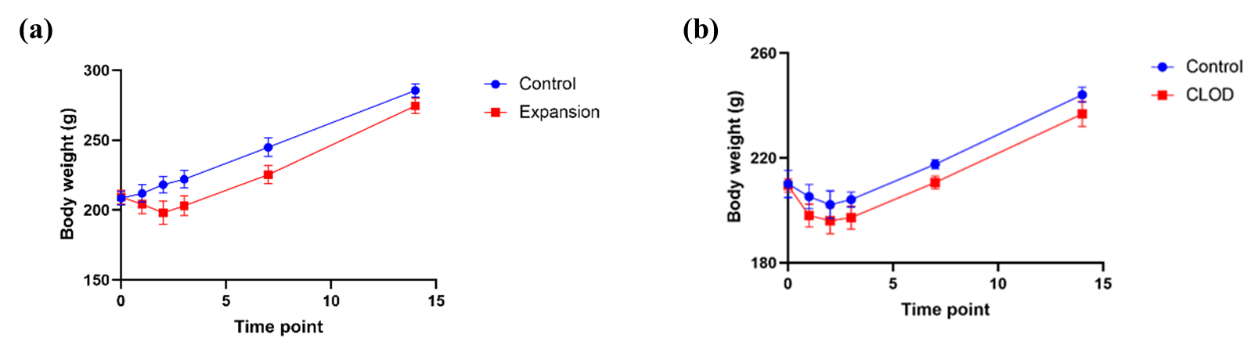
**

**Fig. S1 (a)** Changes in body weight during the experimental period. Body weight curves of control and expansion groups. The weight of the expansion group gradually decreased, reaching the lowest on the third day, after which the weight slowly recovered. The weight of the control group gradually increased over time. **(b)** Changes in body weight after depletion macrophage during the experimental period. Body weight curves of control and CLOD groups. The weight of the two groups gradually decreased, reaching the lowest on the third day, after which the weight slowly recovered.


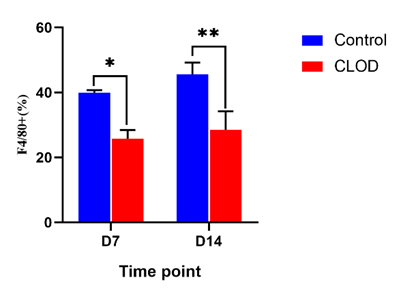


**Fig. S2** Rats were injected with clodronate liposome or PBS by tail vein and killed at days 7 and 14. Flow cytometric analyses of whole bone marrow cells were performed using anti-mouse F4/80 antibodies. The graph shows a quantitative analysis of F4/80+ cells. It can be seen that macrophages were significantly reduced after the injection of clodronate liposomes. **P* < 0.05, ***P* < 0.01.
